# Quorum Sensing Inhibition and Structure–Activity Relationships of β-Keto Esters

**DOI:** 10.3390/molecules21080971

**Published:** 2016-07-25

**Authors:** Stephanie Forschner-Dancause, Emily Poulin, Susan Meschwitz

**Affiliations:** Department of Chemistry, Salve Regina University, 100 Ochre Point Ave, Newport, RI 02840, USA; stephanie.dancause@salve.edu (S.F.-D.); emily.poulin@salve.edu (E.P.)

**Keywords:** quorum sensing inhibition, β-keto esters, *Vibrio harveyi*

## Abstract

Traditional therapeutics to treat bacterial infections have given rise to multi-drug resistant pathogens, which pose a major threat to human and animal health. In several pathogens, quorum sensing (QS)—a cell-cell communication system in bacteria—controls the expression of genes responsible for pathogenesis, thus representing a novel target in the fight against bacterial infections. Based on the structure of the autoinducers responsible for QS activity and other QS inhibitors, we hypothesize that β-keto esters with aryl functionality could possess anti-QS activity. A panel of nineteen β-keto ester analogs was tested for the inhibition of bioluminescence (a QS-controlled phenotype) in the marine pathogen *Vibrio harveyi.* Initial screening demonstrated the need of a phenyl ring at the C-3 position for antagonistic activity. Further additions to the phenyl ring with 4-substituted halo groups or a 3- or 4-substituted methoxy group resulted in the most active compounds with IC_50_ values ranging from 23 µM to 53 µM. The compounds additionally inhibit green fluorescent protein production by *E. coli* JB525. Evidence is presented that aryl β-keto esters may act as antagonists of bacterial quorum sensing by competing with N-acyl homoserine lactones for receptor binding. Expansion of the β-keto ester panel will enable us to obtain more insight into the structure–activity relationships needed to allow for the development of novel anti-virulence agents.

## 1. Introduction

The misuse and overuse of antibiotics in pharmacotherapy and animal production for food have led to the development of widespread resistance and has been cited as a major threat to human and environmental health [1]. The World Health Organization has recently categorized multi-drug resistant bacteria as one of the top three threats to human health [2]. The failure of existing antibiotics to control infection makes it crucial to find alternatives to currently available drugs, particularly ones that do not impose harsh selective pressure for bacteria to develop resistance.

Bacterial cell–cell communication, commonly known as quorum sensing (QS), synchronizes the behaviors of individual cells within a multicellular community [3]. This communication relies upon the production, release, and detection of small diffusible signal molecules termed autoinducers. A two-component system is responsible for QS in many gram negative bacteria and consists of the synthesis of the autoinducer by LuxI homologues and the autoinducer-dependent transcriptional activator, a LuxR homologue [4]. When the autoinducers reach a threshold concentration, the local population of bacteria is able to coordinate the expression of specific genes.

Many pathogenic bacteria use QS to aid in pathogenesis through the simultaneous production of virulence factors necessary for host infection, or to evade the immune system by coordinating swarming behavior and/or biofilm production [4,5]. Hence, interference with bacterial communication has become an attractive target for the development of novel therapeutics in medicine and in the agriculture and aquaculture industries. Such anti-virulence drugs, which target pathways not essential for growth, are hypothesized to lead to a lower spread of resistance [6] and could help to ease the threat of antibiotic resistance.

**Figure 1:**
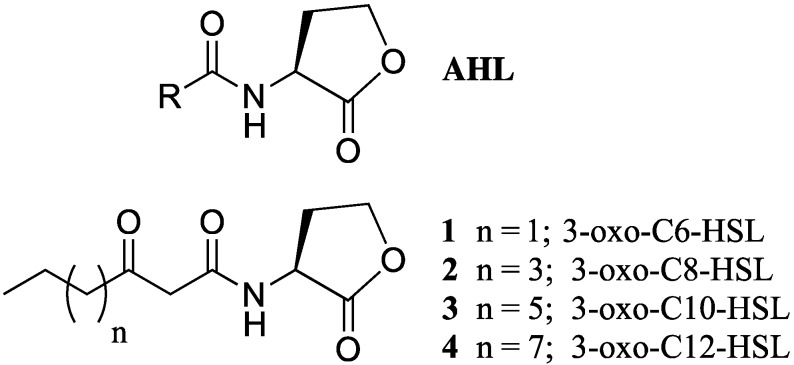
Generic structure for *N*-acylated homoserine lactones (AHLs) and structures of select native AHL ligands containing the β-keto (3-oxo) moiety.

**Figure 2:**
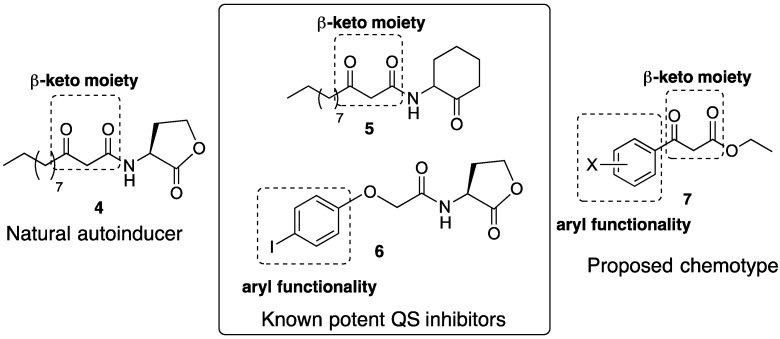
Natural autoinducer employed by *P. aeruginosa* (**4**, **left**); Synthetic AHL-based inhibitors of LasR-dependent quorum sensing, reported by Smith and co-workers [16] and Blackwell and co-workers [22]; **5** and **6** respectively (**center**); Our proposed QS inhibitor chemotype (**7**, **right**).

Synthetic mimics of natural substrates have proven to be promising starting points for designing inhibitors of bacterial QS. Non-natural derivatives of the *N*-acylated homoserine lactone (AHL)—the most common autoinducers used by gram negative bacteria [7]—have shown promise for disrupting QS [8,9]. In contemplating new scaffolds that could inhibit QS pathways, we were intrigued by the β-keto moiety (3-oxo) present in many native AHLs (Figure 1), including the 3-oxo-C6-homoserine lactone (HSL) (**1**) of *Vibrio fischeri* [10] and the pathogen *Erwinia carotovora* [11], the 3-oxo-C8-HSL (**2**) of *Agrobacterium tumefaciens* [12], the 3-oxo-C10-HSL (**3**) of enteric pathogen *V. fluvialis* [13], and the 3-oxo-C12-HSL (**4**) of *Pseudomonas aeruginosa* [14]. However, hydrolysis of the lactone present in the AHLs by mammalian lactonases [15] limits their potential as anti-virulence drugs. Several groups have identified non-natural modulators of AHL-based quorum sensing in which the native homoserine lactone moiety has been replaced with an aromatic group or with cyclic carbocycles (**5**, Figure 2) [5,16,17]. It has also been shown that the central amide connective function of AHLs can be replaced with various non-native moieties, and these non-natural derivatives still retain activity as synthetic modulators of LuxR-based quorum sensing [18,19,20]. In addition, previous results from the literature demonstrate that the incorporation of aryl functionality with electron-withdrawing groups onto the acyl side chain renders many small-molecule AHL mimics potent quorum sensing inhibitors (**6**, Figure 2) [21,22,23]. Thus, we hypothesize that the simplest structural motif that could possess anti-QS activity might be β-keto esters containing aryl functionality (**7**, Figure 2).

## 2. Results and Discussion

**Figure 3:**
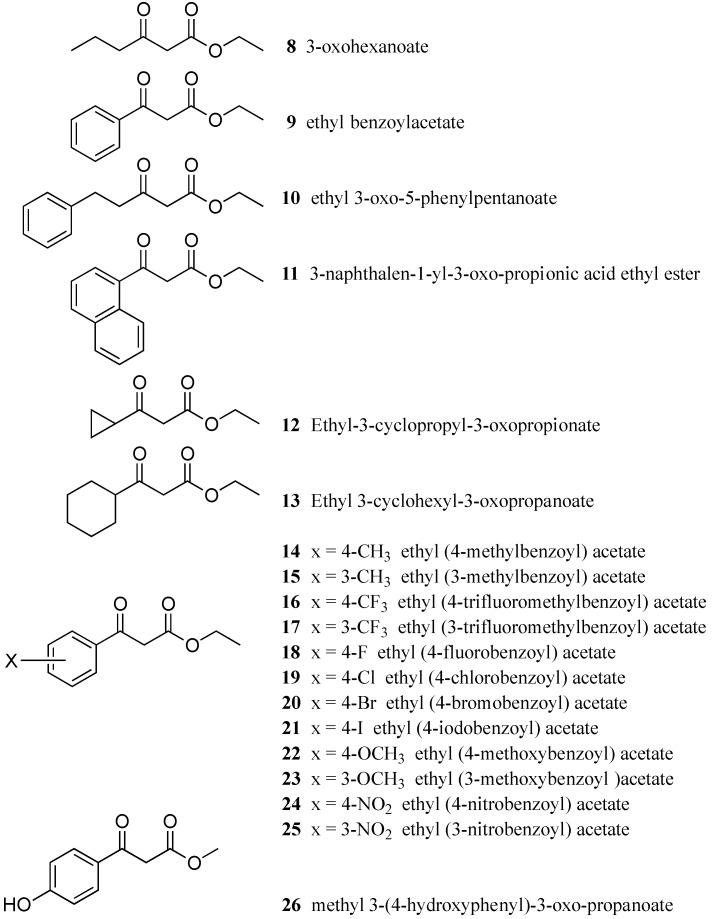
Chemical structures of β-keto ester library.

To investigate the hypothesis, a panel of 19 analogs was tested for the inhibition of bioluminescence—a QS controlled phenotype—in *Vibrio harveyi* (Figure 3). *V. harveyi* and closely related species are one of the most common and serious pathogens in fish and shellfish marine aquaculture worldwide. In vivo studies of QS inhibitors have shown protection of marine organisms against *V. harveyi* infection, thus demonstrating their promise as bacterial disease control agents [24]. Initial screening of a small panel of β-keto esters was accomplished using a disk diffusion assay using the QS reporter strain *V. harveyi* BB120, a wild-type bioluminescence strain [25]. Bioluminescence in *V. harveyi* BB120 is under the control of three distinct QS autoinducers; an AHL, the universal autoinducer 2, and the cholerae autoinducer 1 [26]. Inhibition of any of the three channels will lead to reduced luminescence in vitro.

The initial β-keto esters tested included ethyl 3-oxohexanoate (**8**), ethyl benzoylacetate (**9**), ethyl 3-oxo-phenylpentanoate (**10**), and 3-naphthalen-1-yl-3-oxo-propionic acid ethyl ester (**11**). Only the ethyl benzoylacetate demonstrated QS inhibition with a zone of luminescence inhibition 27 mm in diameter and with no visible inhibition of growth.

Since initial screening indicated the importance of the phenyl ring for antagonist activity, the panel of β-keto esters was expanded to include thirteen additional analogs with varying substituents on the aromatic ring (Figure 3). The subsequent dose–response assays were performed in broth to allow for quantification of luminescence [25] and determination of IC_50_ values (Table 1). Initially, the original four alkyl or aryl-substituted β-keto esters (**8–11**) were evaluated in the dose–response broth assay. The bacterial natural product, 3-methyl-*N-*(2′-phenylethyl)-butyramide, was previously reported to have an IC_50_ of 44 µM in *V. harveyi* BB120 [25]. The compound was used as a control and experiments yielded a comparable IC_50_ of 48 µM. The alkyl compound **8** showed no antagonistic activity, confirming the results of the disk diffusion assay, while the benzoyl compound **9** inhibited 50% of the control *V. harveyi* BB120 bioluminescence at a concentration of 76 μM. By moving the phenyl ring two carbons further away from the 3-oxo moiety in compound **10**, the antagonist activity dropped significantly, suggesting a steric hindrance limitation. The addition of the bulky naphyl group in compound **11** was slightly less active than the benzoyl compound **9**, suggesting that a bulky group at the C-3 position of the β-keto ester is important in inhibiting QS in *V. harveyi.* However, extending the bulky group to a position further from the C-3 to the C-5 position results in a loss of activity. These results may also suggest that π-π interactions between the aromatic ring and aromatic amino acids in the receptor may be important for activity. Further evidence for the importance of such interactions is demonstrated by the inactivity of bulky groups that lack aromaticity, such as the cyclopropyl (**12**) and cyclohexyl (**13**) compounds.

Simple carbon substituents on the benzoyl compound **9** were investigated to probe for structure–activity relationships related to the position of substituents on the aromatic ring, as well as additional steric and electronic limitations. A 4-methyl substituted benzoyl compound (**14**) had slightly less antagonist activity when compared to the non-substituted benzoyl compound **9**. However, moving the methyl substituent to the 3 position of the phenyl ring (**15**) resulted in an IC_50_ of 56 μM. Increasing the bulk of the methyl substituent by replacing it with a 4- or 3-substituted trifluoromethyl (**16** and **17**, respectively) resulted in inhibition similar to the 4-methylbenzoyl compound (**14**). 

Addition of halo substituents to the phenyl ring also resulted in increased antagonist activity over the non-substituted compound (**9**). An IC_50_ of 23 μM was obtained for the 4-fluoro compound (**18**). Antagonist activity then decreased with the increasing atomic size of the 4-chloro (**19**) and 4-bromo (**20**) compounds. Surprisingly, the trend does not continue with the 4-iodo compound (**21**), and instead, the antagonist activity increases to an IC_50_ of 39 μM. The 4-fluoro and 4-iodo substituted benzoyl compounds represent two of the three strongest inhibitors of QS in this study. While only the 4-substituted position of the halogens was investigated, it would be interesting to see the effect 3-substituted halogens would have on the activity, considering the results of the 3-methyl benzoyl compound (**15**).

The influence of the position of other heteroatom substituents on the aromatic ring of the β-keto ester ethyl benzoylacetate (**9**) were evaluated, however no consistent trends emerged. The 4- and 3-substituted methoxy compounds (**22** and **23**, respectively) showed strong antagonist activity, with the 4-methoxy analogue having a slightly lower IC_50_ of 36 μM to an IC_50_ of 41 μM for the 3-methoxy analogue. The same trend was seen in the nitro analogs, with the 4-substituted nitro compound (**24**) having a lower IC_50_ than the 3-substituted nitro compound (**25**, IC_50_ of 53 μM and 71 μM, respectively). The opposite trend was seen with the methyl analogs (**14** and **15**), in which the 3-substituted position was the stronger antagonist. The hydroxyphenyl analogue (**26**) demonstrated no inhibitory activity. It represents the only methyl ester in the panel, and thus may argue the need of an ethyl ester or possibly a larger alky or aryl substituent bound to the oxygen in the ester. When comparing electron donating and electron withdrawing properties within our full panel of β-keto esters, no discernable trends were observed at the 4-position with the substituents tested. However, the 3-position substituted derivatives appear to be more active with the presence of electron donating groups than with electron withdrawing groups.

Growth curves were conducted on all β-keto ester analogs to ensure that the observed inhibition of luminescence was not due to inhibition or delay in growth of *V. harveyi* by the analogs. Only one of the 13 active compounds—the 4-substituted nitro derivative (**24**)—demonstrated a delay in growth at the IC_50_ concentrations (Supplementary Material Figures S15 and S16). The delay in growth due to the 4-NO_2_ derivative is apparent at the 5 h time point in which luminescence is measured, and could account for the observed inhibition—even after normalizing for growth. Additionally, a luminescence curve for the 4-fluoro substituted derivative—the most active compound—demonstrated that the luminescence inhibition persists for the duration of luminescence production by the control (Supplementary Material Figure S17).

**Figure 4:**
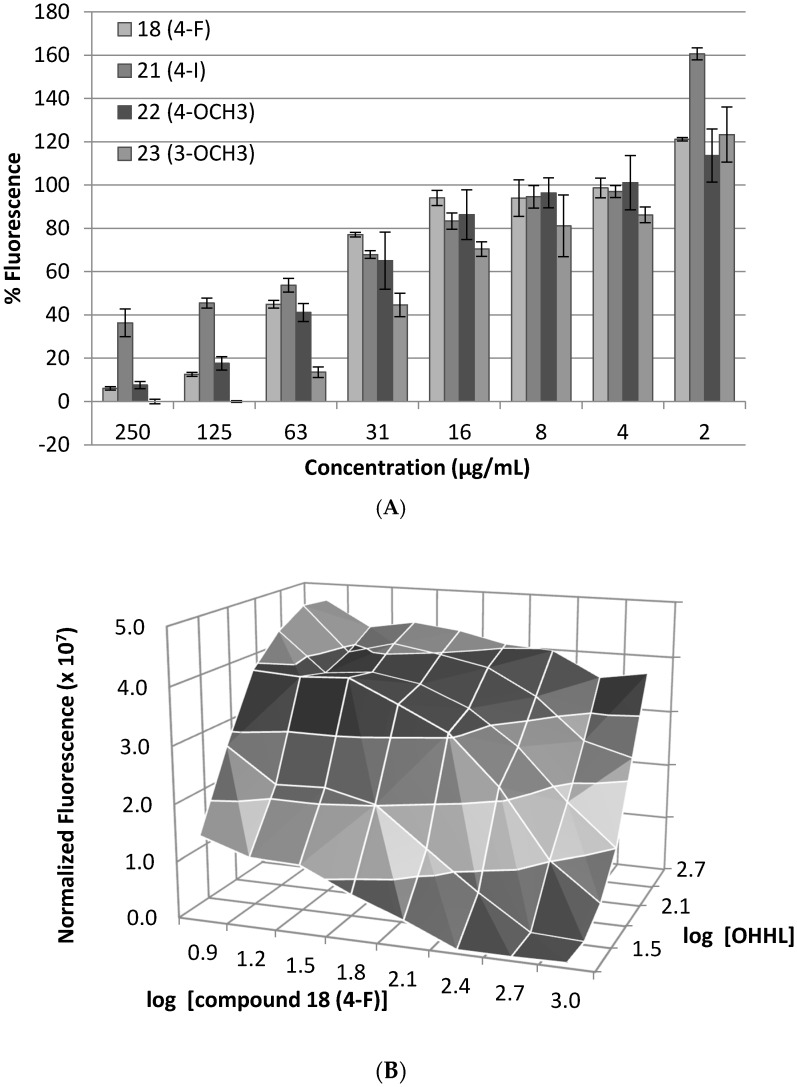
Inhibition of 3-oxo-hexanoyl-homoserine lactone (OHHL)-mediated green fluorescent protein (GFP) production in *E. coli* JB525 by various β-keto ester analogs. (**A**) Concentration-dependent inhibition of GFP production (fluorescence) by select β-keto ester analogs augmented with 32 nM OHHL, in relation to the GFP production of *E. coli* JB525 with 32 nM OHHL only (defined as 100% fluorescence). Error bars represent standard deviation of three biological replicates; (**B**) Surface 3D plot of GFP production (fluorescence normalized by growth, OD_450_) at various concentrations of antagonist (compound **18**, µM) and agonist (OHHL, nM). Inhibition by compound **18** is overcome by increasing concentrations of OHHL, consistent with competitive inhibition.

The panel of β-keto ester analogs demonstrated strong QS antagonist activity against the wild-type reporter strain *V. harveyi* BB120. Though the exact mechanism is not clear with this assay, the structural similarities to AHL autoinducers suggests that the β-keto ester could modulate LuxR-based QS through the AHL-dependent pathway. The mutant *Escherichia coli* JB525, containing the *luxR*-P*_luxI_* from *V. fischeri* fused to *gfp*, was used to evaluate the mechanism of QS inhibition [27]. The biosensor produces unstable green fluorescent protein (GFP) in response to the activation of LuxR by exogenous AHL. Binding of an inhibitor to LuxR in the presence of exogenous AHL will lead to reduced GFP, suggesting that the inhibitor and AHL compete for the same receptor [28]. A dose–response competition assay was performed on the β-keto ester analogs with IC_50_ values less than 50 µM, while holding the concentration of the native *V. fischeri* AHL—3-oxo-hexanoyl-HSL (**1**, OHHL)—at 32 nM. All four showed concentration-dependent inhibition of GFP, suggesting antagonistic activity of AHL-mediated QS (IC_50_ values: 4-flouro (**18**) = 48 µg/mL, 4-iodo (**21**) = 88 µg/mL, 4-methoxy (**22**) = 45 µg/mL, and 3-methoxy (**23**) = 24 µg/mL; Figure 4a). The bacterial natural product 3-methyl-*N-*(2′-phenylethyl)-butyramide was used as a control, and an IC_50_ of 29 µg/mL was obtained, compared to the reported IC_50_ of 19 µg/mL [25]. No growth inhibition of *E. coli* JB525 was observed at the concentrations tested.

To further probe the mechanism of fluorescence inhibition, the 4-fluoro substituted analog (**18**) was tested against increasing concentrations of OHHL (16 to 512 nM). As the OHHL concentration increased, the effect of the antagonist diminished (Figure 4b). These results suggest that these β-keto esters are competitive antagonists of AHLs in LuxR-mediated QS.

## 3. Experimental

### 3.1. Test Compounds

The β-keto ester compounds were purchased from Sigma Aldrich (St. Louis, MO, USA) and purity was verified by ^1^H-NMR and HPLC analysis (Supplementary Material Figures S1–S14). 3-methyl-*N*-(2′-phenylethyl)-butyramide was synthesized as reported previously [25] and used as a positive control in broth dilution assays. The 3-oxo-hexanoyl-homoserine lactone (OHHL) was purchased from Sigma Aldrich.

### 3.2. Disc Diffusion Bioassay

*V. harveyi* BB120 was grown at 30 °C in yeast peptone (YP) media (0.1% *w/v* yeast extract, 0.5% *w/v* peptone, and 2.25% *w/v* instant ocean). Sterile 6 mm disks were loaded with 400 μg of test compound and placed atop a 6 mL semi-soft YP agar (0.8% *w/v* agar) lawn seeded with 60 μL of overnight bacterial culture prior to being poured on a YP agar (1.5% *w/v* agar). The plates were incubated at 30 °C for 18–24 h, and zones of luminescence inhibition were measured using the G:Box Chemi xx6 (Syngene, Cambridge, UK).

### 3.3. Dose–Response Bioassay

To allow for quantification of luminescence, dose–response assays were performed in broth [25]. An overnight culture of *V. harveyi* BB120 was first diluted in YP to an optical density of 0.1 at 600 nm. The initial dilution was subsequently diluted 50-fold, and 199 μL was added to each well of a 96-well opaque wall, clear bottom microtiter plate. Test compounds were brought up to a concentration of 0.2 M in DMSO, and 2-fold serial dilutions were performed to obtain a total of eight concentrations. *V. harveyi* is sensitive to DMSO, and therefore 1 μL of each dilution was added to the 199 μL of bacterial cultures in the well to keep the final DMSO concentration at 0.5%. Each compound was tested in three biologically separate replicates, and control wells contained the bacterial culture with 0.5% of DMSO (no compound). The plates were incubated at 30 °C with shaking for 5 h. Luminescence and optical density at 600 nm were read on a SpectraMax i3 multi-mode microplate reader (Molecular Devices, Sunnyvale, CA, USA). Luminescence was normalized by optical density, and percent luminescence was calculated by defining the control wells as 100%. Compounds with an IC_50_ bioluminescence value greater than 100 µM were considered inactive. To determine minimum inhibitory concentrations for growth, the plates were allowed to incubate overnight. Growth and luminescence curve experiments were conducted as described above at each active compound’s IC_50_ or 250 µM, respectively. The OD_600_ or luminescence was read hourly for 24 h to investigate possible temporal growth effects and duration of luminescence inhibition. 

### 3.4. Competitive Antagonism Bioassay

The mutant *E. coli* JB525 (*E. coli* MT102 containing the recombinant plasmid pJBA132) was used to investigate AHL-dependent QS inhibition [27]. The mutant links GFP production to activation of *V. fischeri* LuxR by exogenously-added AHLs, especially OHHL. Antagonism of LuxR was determined using methods described in Teasdale, et al. [25]. Briefly, *E. coli* JB525 was grown at 30 °C in LB broth containing 4 g sodium chloride. Overnight cultures were diluted to an OD_450_ of 0.1. Cultures were treated with 32 nM OHHL and test compound ranging from 2 to 250 µg/mL (0.5% final DMSO concentration in 200 µL). To determine antagonist–agonist relationships, each serial dilution of test compound (8–1000 µM) was challenged with each increasing OHHL concentration (16–512 nM) in three biologically separate replicates (0.7% DMSO final concentration). Plates were incubated with shaking at 30 °C for 90 m. Fluorescence was detected with an excitation at 475 nm and emission at 515 nm on the SpectraMax i3 multi-mode microplate reader (Molecular Devices, Sunnyvale, CA, USA). Growth was evaluated after 90 min by optical density at 450 nm. Fluorescence values were normalized by optical density. For IC_50_ determination at 32 nM OHHL, percent fluorescence was determined by defining control wells with 32 nM OHHL as 100% fluorescence. 

## 4. Conclusions

In conclusion, a panel of β-keto ester analogs was examined for the ability to inhibit quorum sensing-regulated luminescence in the reporter strain *V. harveyi* BB120. The four most active derivatives included the aryl substituted analogs 4-flouro (**18**), 4-iodo (**21**), 4-methoxy (**22**), and 3-methoxy (**23**), with percent luminescence ranging from 6.5% to 34% at 125 µM with no inhibition of growth. The β-keto ester analogues have structural and/or physical features similar to that of the AHL autoinducer, and additional assays with an *E. coli* biosensor JB525 suggest that the active analogs interact with the Lux-R-type proteins to inhibit QS. Expansion of the panel through structural modification will enable us to obtain more insight into the structure–activity relationships needed to allow for the development of novel anti-virulence agents.

## Figures and Tables

**Table 1:** Inhibitory concentration of 50% bioluminescence after 5 h and minimum growth inhibitory concentration after 24 h in *V. harveyi*. IC_50_ values greater than or equal to 100 µM were considered inactive.

Compound	IC_50_ (µM)	MIC (µM)	Compound	IC_50_ (µM)	MIC (µM)
8	inactive	>1000	18	23	>1000
9	76	>1000	19	53	1000
10	inactive	>1000	20	81	500
11	87	>1000	21	39	500
12	inactive	>1000	22	36	>1000
13	inactive	>1000	23	41	>1000
14	96	>1000	24	53	>1000
15	56	>1000	25	71	>1000
16	inactive	1000	26	inactive	>1000
17	92	1000			

## Supplementary Materials

Supplementary materials can be accessed at: www.mdpi.com/1420-3049/21/8/971/s1.

## Abbreviations

The following abbreviations are used in this manuscript:

QS	Quorum sensing
AHL	*N*-acylated homoserine lactone
HSL	Homoserine lactone
MIC	Minimum inhibitory concentration
GFP	green fluorescent protein
OHHL	3-oxo-hexanoyl-HSL
YP	yeast peptone media
