# Supplementary material for: Quorum Sensing Inhibition and Structure–Activity Relationships of β-Keto Esters
Source: Molecules. 2016 Jul 25;21(8):971. doi: 10.3390/molecules21080971 (PMC5771494; doi:10.3390/molecules21080971)
Supplement: Supplementary file 1 [file molecules-21-00971-s001.pdf]

Supplementary Materials: Quorum Sensing Inhibition and Structure Activity Relationships of β-Keto Esters

Stephanie Forschner-Dancause, Emily Poulin and Susan Meschwitz

HPLC Analysis of β-Keto Esters

The reversed-phase HPLC analysis was carried out using a Phenomenex Luna C18 (250 mm × 4.6 mm, 5 μm) column. A linear gradient chromatographic technique was used at room temperature with the following solvent system for all compounds except **24** and **25**: solvent A = 0.1% trifluoroacetic acid; solvent B = methanol; starting at 65% A:35% B and ending 20 m later with 0% A:100% B and a flow rate set at 1 mL/min. For compounds **24** and **25**, an isocratic mobile phase consisting of water and 2-propanol (50:50 v/v) was used. The detection of compounds was carried out at 254 nm.

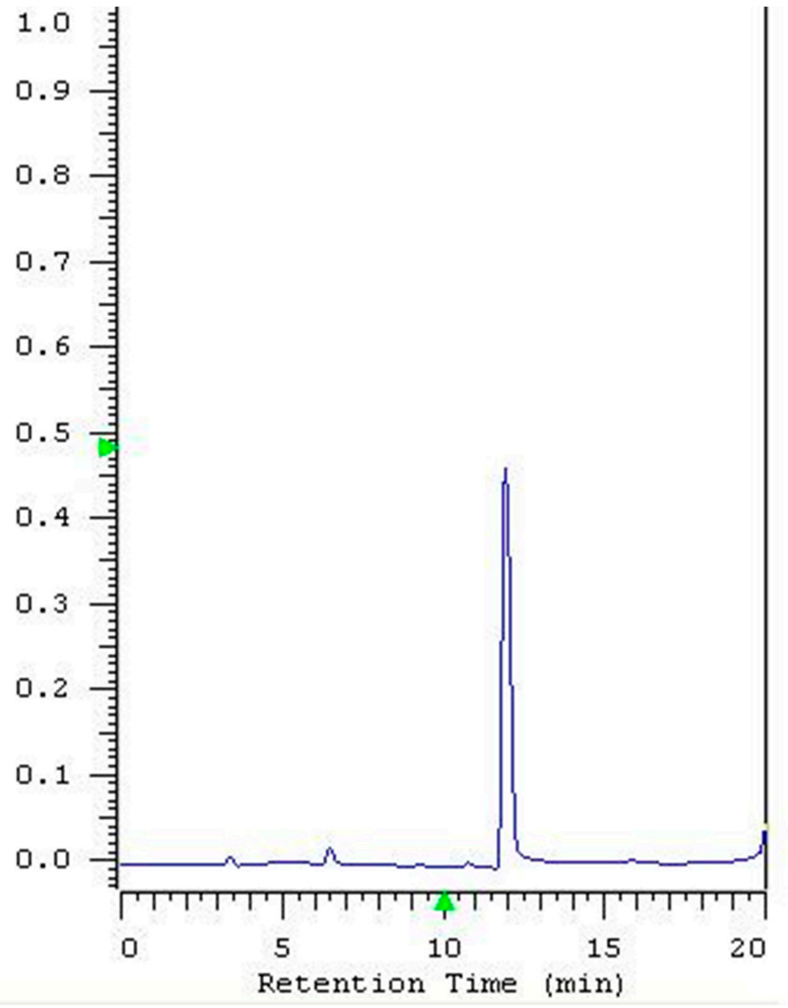

Figure S1. HPLC profile of compound **9** ethyl benzoyl acetate.

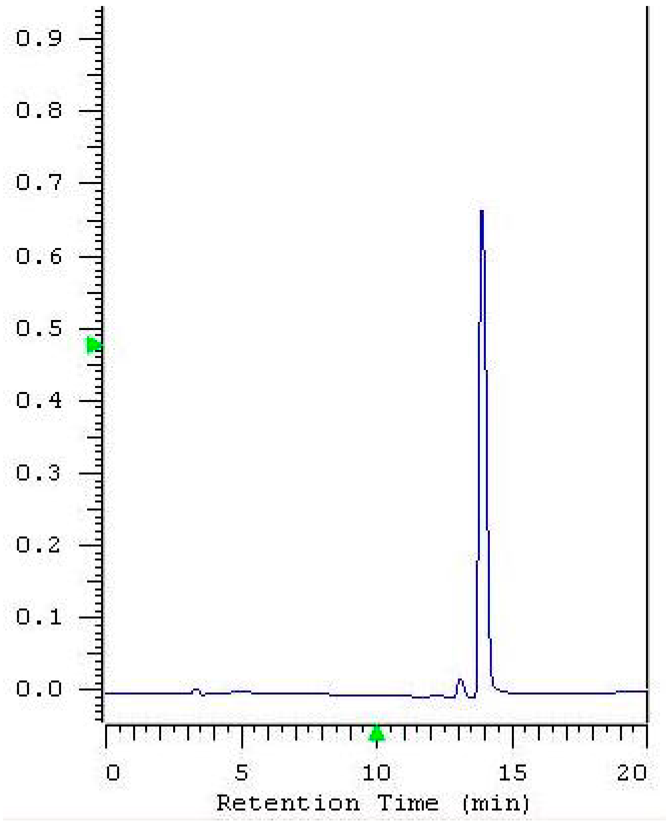

Figure S2. HPLC profile of compound **14** ethyl (4-methylbenzoyl) acetate.

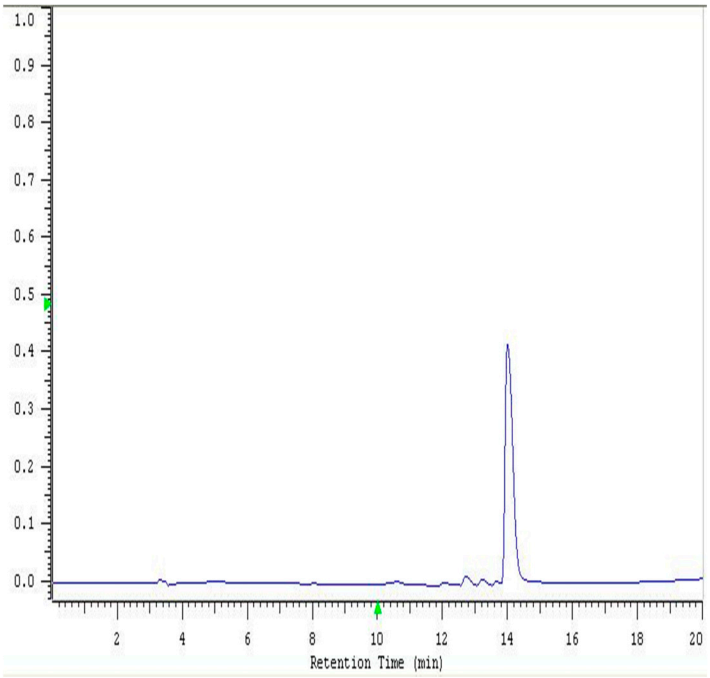

Figure S3. HPLC profile of compound **15** ethyl (3-methylbenzoyl) acetate.

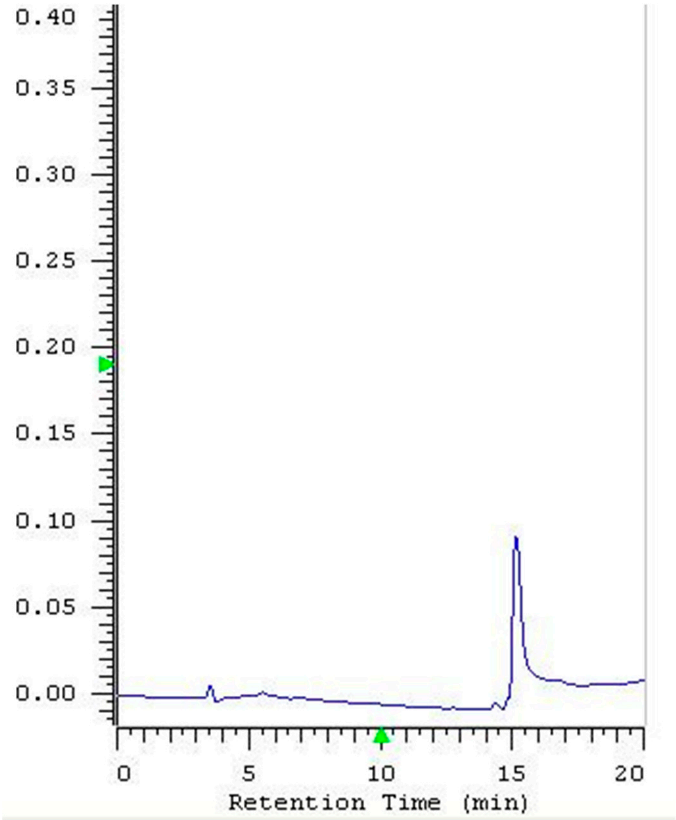

Figure S4. HPLC profile of compound **16** ethyl (4-trifluoromethylbenzoyl) acetate.

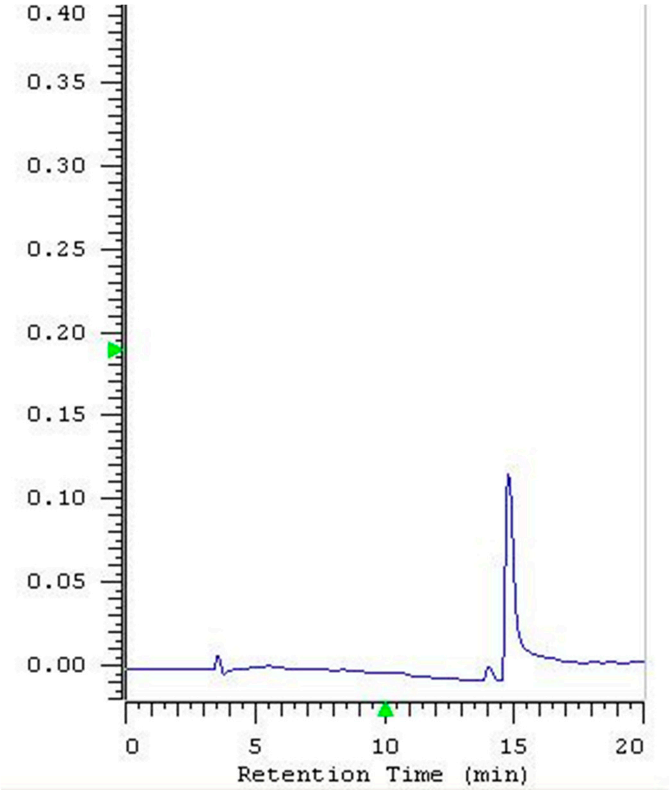

Figure S5. HPLC profile of compound **17** ethyl (3-trifluoromethylbenzoyl) acetate.

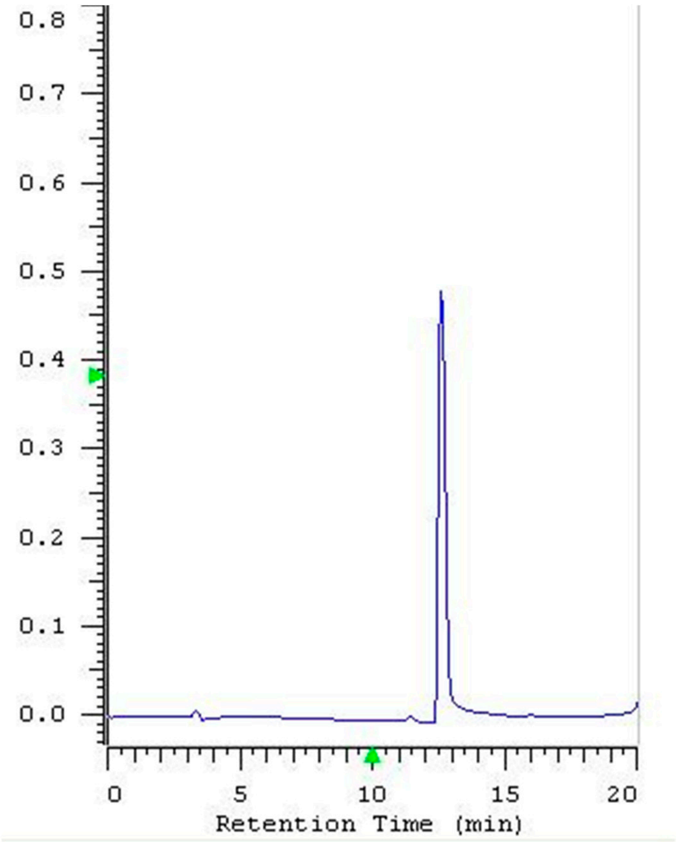

Figure S6. HPLC profile of compound **18** ethyl (4-fluorobenzoyl) acetate.

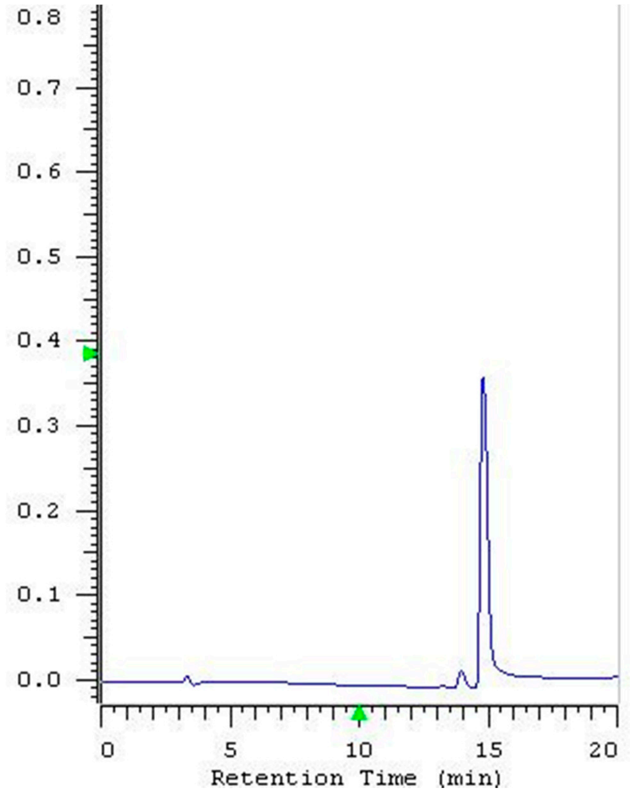

Figure S7. HPLC profile of compound **19** ethyl (4-chlorobenzoyl) acetate.

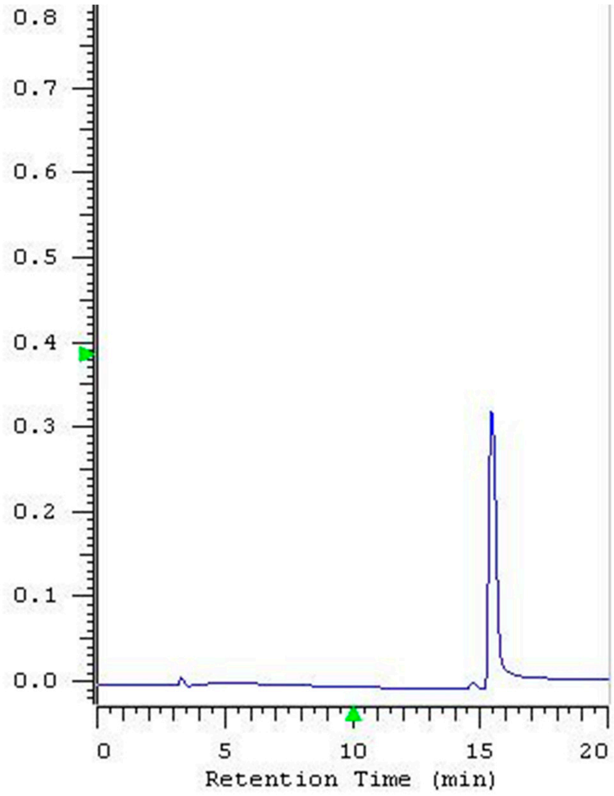

Figure S8. HPLC profile of compound **20** ethyl (4-bromobenzoyl) acetate.

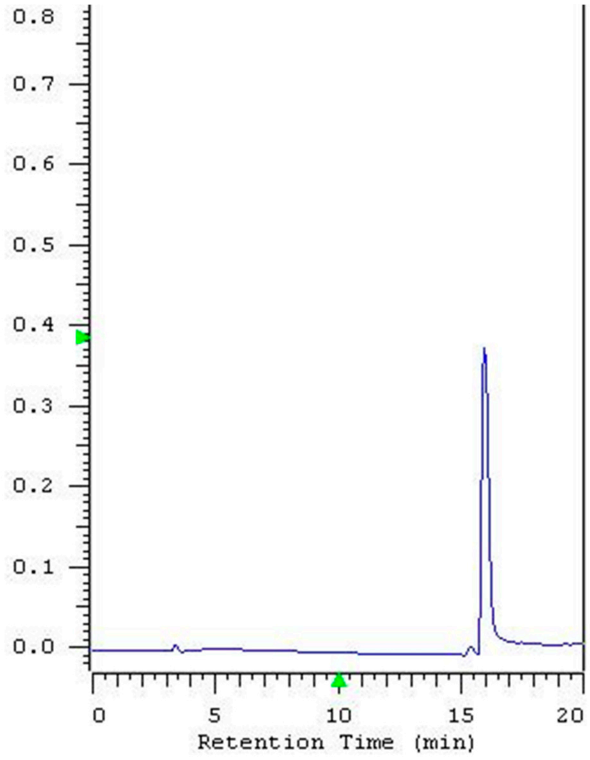

Figure S9. HPLC profile of compound **21** ethyl (4-iodobenzoyl) acetate.

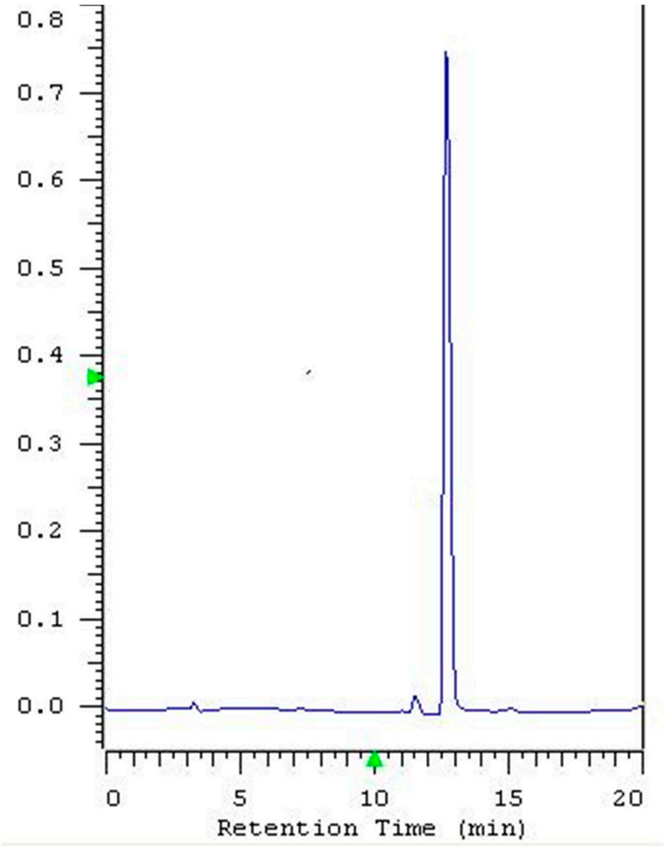

Figure S10. HPLC profile of compound **22** ethyl (4-methoxybenzoyl) acetate.

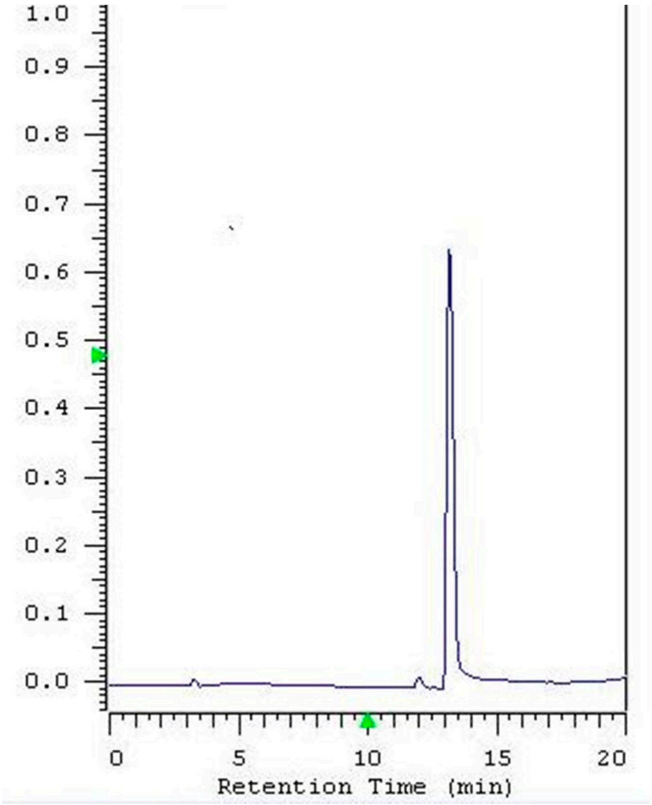

Figure S11. HPLC profile of compound **23** ethyl (3-methoxybenzoyl) acetate.

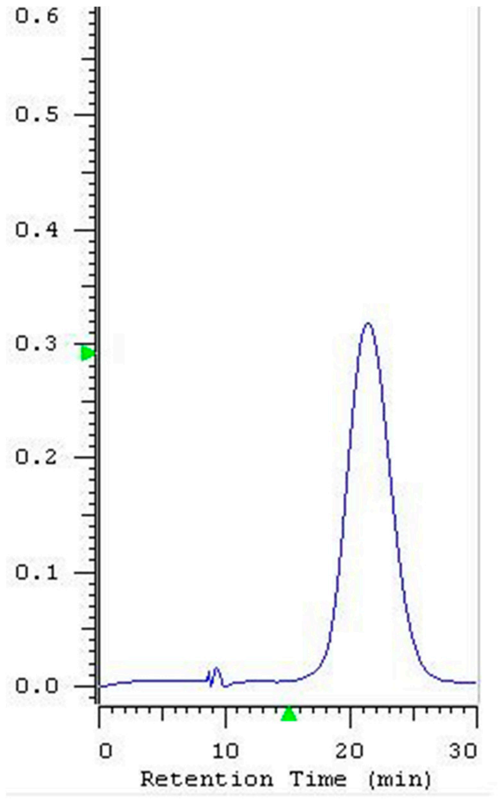

Figure S12. HPLC profile of compound **24** ethyl (4-nitrobenzoyl) acetate.

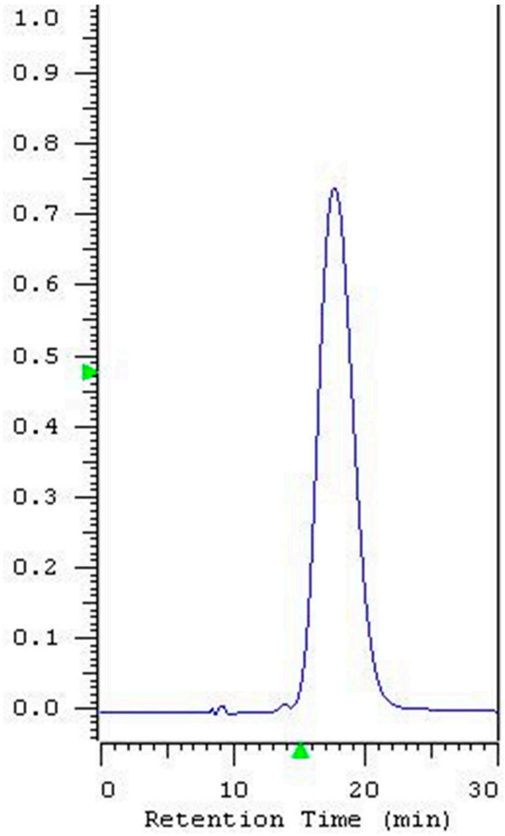

Figure S13. HPLC profile of compound **25** ethyl (3-nitrobenzoyl) acetate.

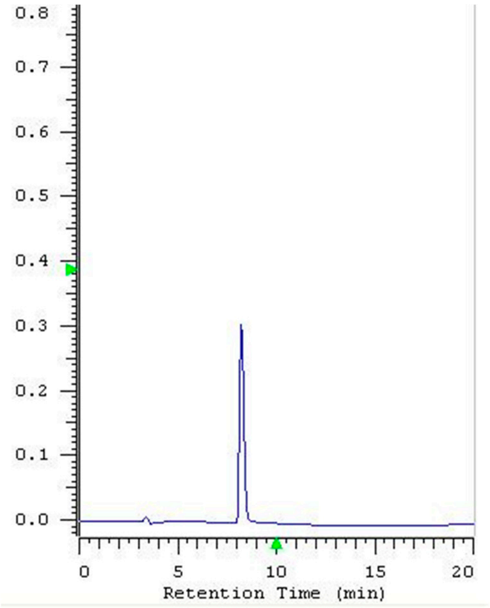

Figure S14. HPLC profile of compound **26** methyl 3-(4-hydroxyphenylyl)-3-oxo-propanoate.

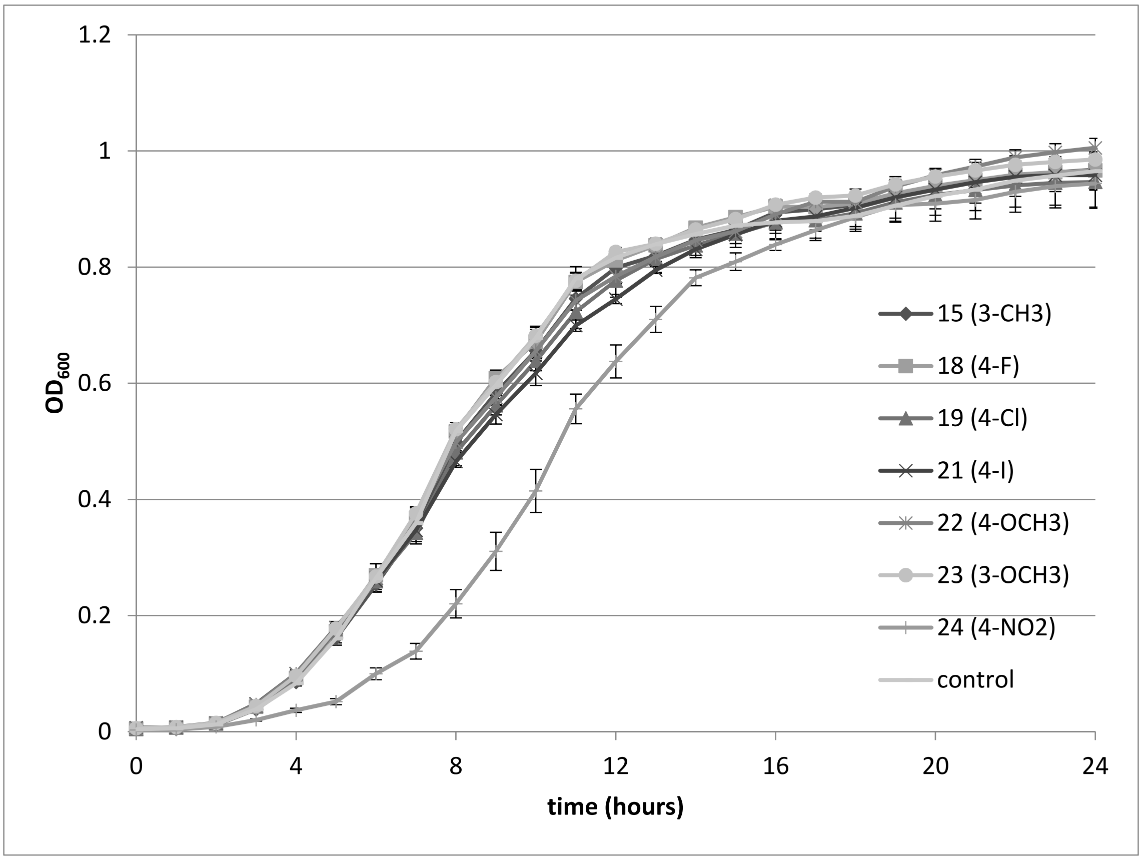

Figure S15. Growth of *V. harveyi* BB120 monitored by optical density at 600 nm for 24 h. Cultures were treated with active β-keto ester analogs at their IC_{50} concentrations. Control contains same concentration of DMSO as treatment but with no added compound. Error bars represent standard deviation of replicates.

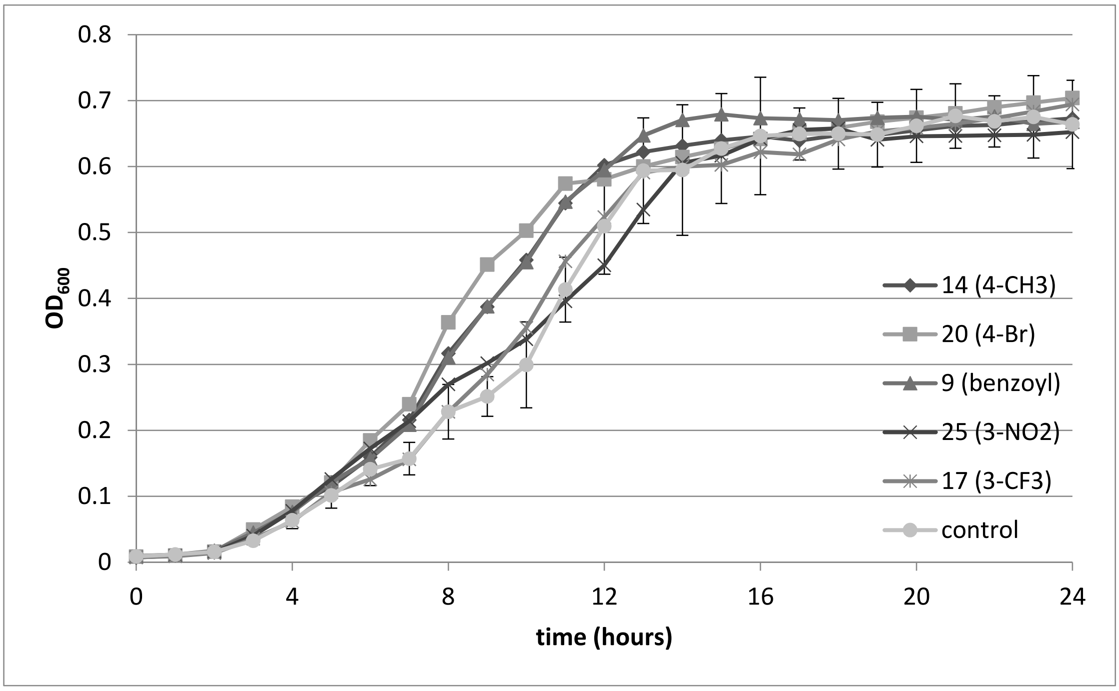

Figure S16. Growth of *V. harveyi* BB120 monitored by optical density at 600 nm for 24 h. Cultures were treated with active β-keto ester analogs at their IC_{50} concentrations. Control contains same concentration of DMSO as treatment but with no added compound. Error bars represent standard deviation of replicates and are shown for control only to simplify figure.

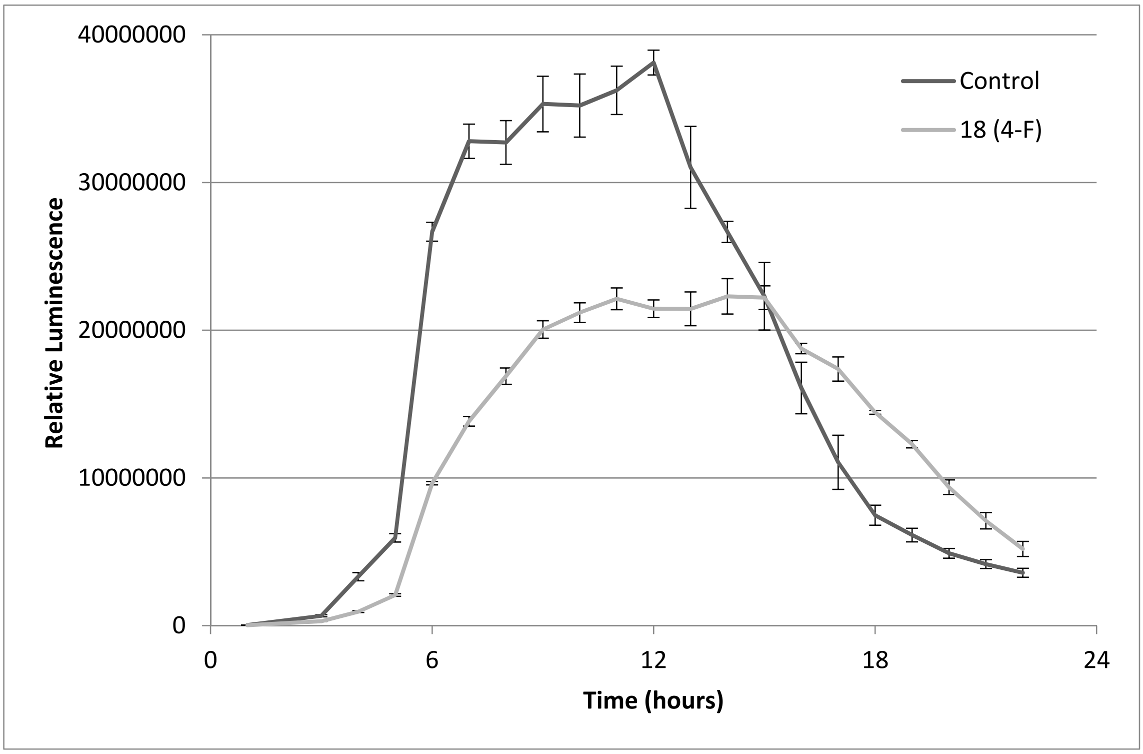

Figure S17. Luminescence by *V. harveyi* BB120 with and without 250 μM ethyl (4-fluorobenzoyl) acetate (**18**). Control contains same concentration of DMSO as treatment but with no added compound. Error bars represent standard deviation of replicates.
